# Retraction: A deep learning model for three-dimensional nystagmus detection and its preliminary application

**DOI:** 10.3389/fnins.2023.1358381

**Published:** 2023-12-28

**Authors:** Frontiers Editorial Office

The journal retracts the 13 June 2022 article cited above.

Following publication, the publisher uncovered evidence that false identities were used in the peer-review process. The assignment of fake reviewers was confirmed by an investigation, conducted in accordance with Frontiers' policies and the Committee on Publication Ethics (COPE) guidelines. Given the concerns, the editors no longer have confidence in the findings presented in the article. UPDATE (30 July 2024): This notice is to alert readers of this matter, it does not imply involvement of the co-authors.

This retraction was approved by the Chief Editors of Frontiers in Neuroscience and the Chief Executive Editor of Frontiers. The authors do not agree to this retraction.

